# A Case Report of a Complete Removable Denture Designed to Restore Function in a Patient With Severe Resorption of the Mandibular Alveolar Ridge Using the Neutral Zone and Flange Techniques

**DOI:** 10.1155/crid/6174765

**Published:** 2026-10-13

**Authors:** Hitoshi Akiyama

**Affiliations:** ^1^ Nippon Dental University Hospital Tokyo Japan

**Keywords:** clinical report, complete denture, flange technique, neutral zone technique, ridge resorption

## Abstract

Edentulous patients with severe mandibular alveolar ridge resorption struggle with denture stability, which often reduces their quality of life due to mucosal pain and food debris retention. When the inferior alveolar canal is extremely close to the alveolar crest, conventional dentures risk causing severe pain and sensory abnormalities by compressing the inferior alveolar nerve. Thus, conventional fabrication methods face clinical limitations. To stabilize a mandibular complete denture in such cases, it is highly effective to record the dynamics of the denture space and fabricate a prosthesis that harmonizes with these perioral muscle movements. A 77‐year‐old woman presented with frequent stomatitis caused by ill‐fitting dentures and severe masticatory dysfunction. Intraoral and radiographic examinations revealed marked resorption of the mandibular alveolar ridge, placing the inferior alveolar canal in extreme proximity to the alveolar crest. The treatment plan involved fabricating a new maxillary partial denture that strictly balanced the support of the remaining teeth. For the mandible, a complete denture was fabricated using the neutral zone and flange techniques to harmonize with surrounding soft tissue movements. This approach enabled functional contouring by leveraging the dynamic pressure of the perioral muscles. Consequently, denture retention and stability improved dramatically, and denture displacement during speech was successfully suppressed. An evaluation of masticatory function confirmed objective functional recovery. This case demonstrates that for patients with severe alveolar ridge resorption and diminished anatomical supporting tissues, determining the functional form of the denture by accounting for soft‐tissue dynamics is highly effective in restoring stability and masticatory function.

## 1. Introduction

In patients with complete edentulism and significant mandibular alveolar bone resorption, achieving adequate denture retention and stability presents significant challenges. This frequently results in mucosal pain and food impaction, reducing quality of life (QOL). Particularly when the mandibular alveolar canal and alveolar ridge are extremely close, conventional denture design can directly compress the mandibular alveolar nerve, increasing the risk of pain and sensory abnormalities.

In edentulous cases with severe alveolar ridge resorption, it is often difficult to achieve sufficient denture stability using conventional impression‐taking methods alone. In particular, perioral muscle activity can cause denture mobility, leading to mucosal pain and food debris retention. Prosthetic rehabilitation for complete denture patients should aim not only at replacing missing teeth, but also at fully restoring masticatory functions, phonetics, and overall facial esthetics [1]. To address this, it is necessary to restore denture stability and function using specialized techniques, such as the neutral zone [2, 3] and flange techniques [4]. To achieve stable prostheses in patients with severe bone resorption, it is crucial to recognize and utilize the forces resulting from muscle function to promote denture stabilization. This can only be executed through a clear understanding of the neutral zone, which is shaped by the perioral musculature including the buccinator, modiolus, tongue, and orbicularis oris muscles [5].

To ensure the stability of a complete lower denture, it is effective to record the dynamics of the denture space and fabricate a denture that harmonizes with these dynamics, thereby utilizing lateral pressure as a retention mechanism to the greatest extent possible.

## 2. Case Description

### 2.1. Patient History and Clinical Presentation

A 77‐year‐old female patient visited the general dentistry at The Nippon Dental University Hospital complaining of significant instability and recurrent pain associated with a complete lower denture fabricated 3 years prior. The patient reported an inability to chew solid foods and felt severe embarrassment during conversation due to the denture frequently shifting. Her medical history included controlled essential hypertension. However, there were no other notable findings, and no systemic contraindications for selective minor oral surgery were identified. Examination outside the oral cavity revealed a reduced occlusal vertical dimension and loss of labial support, which contributed to premature facial aging.

#### 2.1.1. Intraoral Findings

Intraoral findings at the initial visit (with the denture removed) are shown in Figure 1. Intraoral clinical examination revealed that the mandibular arch was completely edentulous. The residual maxillary alveolar ridge had adequate height and width, and the mucosal coverage was good. In contrast, the mandibular alveolar ridge showed marked vertical and horizontal atrophy and was characterized as Type V (flat ridge form, inadequate in height and width) in the Cawood and Howell classification [6]. The floor of the mouth was highly mobile, and it was confirmed that when the tongue was elevated, it easily shifted over the crest of the residual alveolar ridge. Regarding the mucosal characteristics, the tissue was soft and thin. Intraoral findings at the initial visit (with dentures in place) are shown in Figure 2. The maxillary denture utilized cast clasps and a palatal bar. The mandibular denture showed wear on the artificial teeth, although occlusal contact was present, wobbling was observed during lateral guidance, indicating poor denture fit.

**Figure 1:**
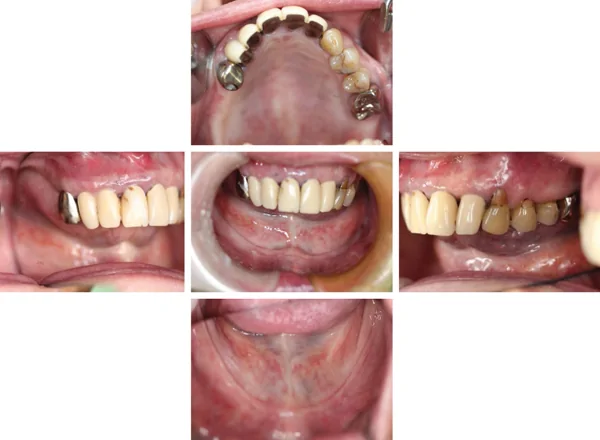
Intraoral findings at the initial visit (without dentures in place).

**Figure 2:**
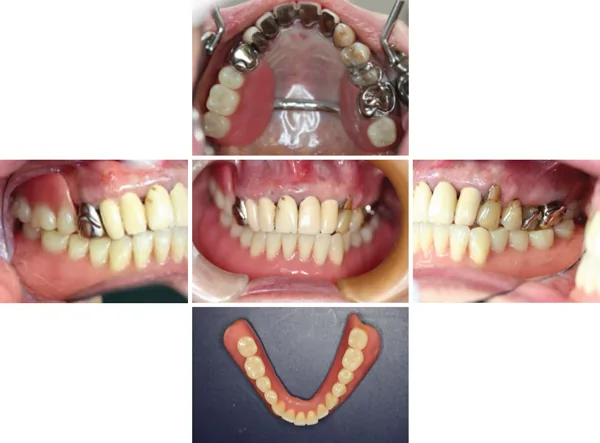
Intraoral findings at the initial visit (with dentures in place).

#### 2.1.2. Radiographic Findings

In the upper jaw, Teeth 14, 13, 12, 11, 21, 22, and 26 show root canal fillings and crown restorations.

Teeth were identified according to the FDI World Dental Federation notation system.

Moderate alveolar ridge resorption is observed in the right‐sided edentulous area. In the lower jaw, marked alveolar ridge resorption resulted in a minimal distance between the inferior alveolar canal and the alveolar crest (Figure 3).

**Figure 3:**
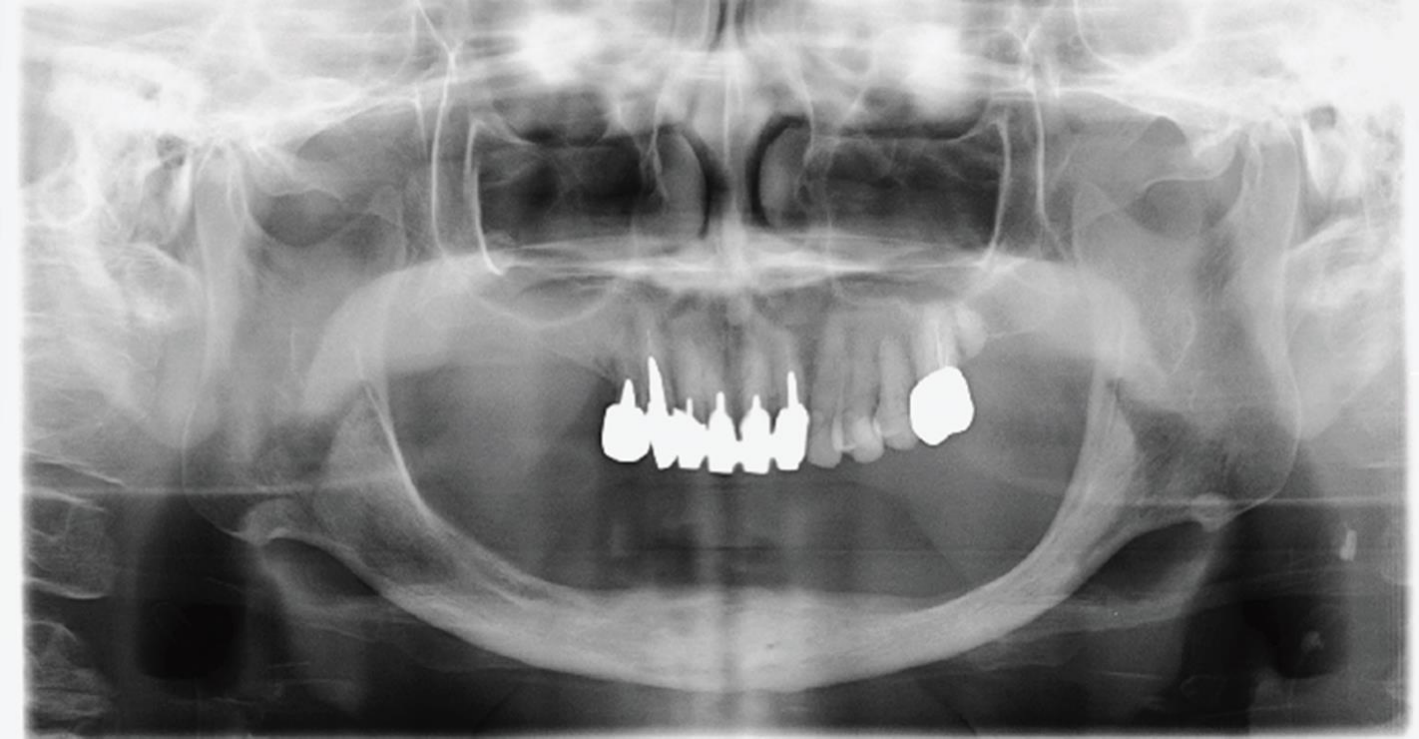
Radiographic findings. Upper jaw: Teeth 14, 13, 12, 11, 21, 22, and 26 show root canal fillings and crown restorations. Moderate alveolar ridge resorption is observed in the right‐sided edentulous area. Lower jaw: Alveolar ridge resorption is marked, and the distance between the inferior alveolar canal and the alveolar crest is minimal.

Masticatory function was evaluated using the Glucosensor GS‐II (GC Corp., Tokyo, Japan). The Glucosensor GS‐II is a glucose analyzer that automatically measures glucose concentration in approximately 6 s by applying a drop of the filtered liquid obtained after chewing a glucose‐containing gummy for 20 s and then spitting it out onto the sensor chip. A glucose concentration below 100 mg/dL is considered to indicate a potential problem with masticatory function [7]. Based on the glucose level in this case, it was 57 mg/dL with the old dentures, indicating that the patient was unable to chew properly.

### 2.2. Treatment Plan and Course of Treatment

Diagnosis: Masticatory dysfunction caused by ill‐fitting dentures due to severe alveolar ridge resorption.

Treatment: After reducing mucosal inflammation by adjusting the old dentures, new dentures were fabricated as follows.

### 2.3. Precision Impression and Occlusal Registration

For the mandible, a primary impression was taken using modeling compound (GC Corp., Tokyo, Japan), followed by a final impression using Iso compound (GC Corp., Tokyo, Japan) and Neodine Impression Paste (NEO Dental Chemical Products Co. Ltd., Tokyo, Japan). Vertical occlusal registration was performed using the mandibular resting position method. The distance between the subnasal point and the mentum was measured five times with calipers. The occlusal vertical dimension was set to 51.5 mm, calculated by subtracting the resting space from the average of the three middle measurements (after excluding the minimum and maximum values). An occlusal registration was then taken using Neodine Impression Paste. To determine the horizontal jaw position, a marking needle was attached to the maxilla and a marking plate to the mandible. The gothic arch marking method was used, and an occlusal impression was taken with Xanthano (Kulzer Japan Co. Ltd., Tokyo, Japan). After the patient practiced the movements several times, the patient was instructed to perform them in the following order: from mandibular retrusion to the anterior, left lateral, and right lateral positions (Figure 4).

**Figure 4:**
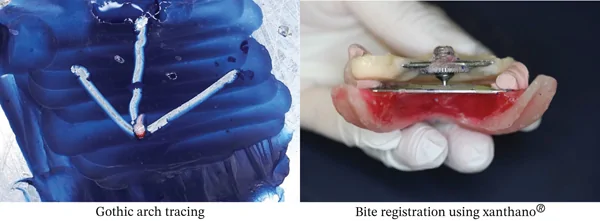
Gothic arch tracing and bite registration.

### 2.4. Design of a Maxillary Partial Denture

The maxillary denture was designed to ensure stable support based on the arrangement of the abutment teeth (14, 25, and 26) (Figure 5). The interproximal lines between the abutment teeth were designed to form a polygon, with the area enclosed by these lines shaped as a triangle. The design was executed with consideration for the load arm and effort arm, using the interproximal lines between the abutment teeth, which serve as the denture′s rotation axis, as the basis. Furthermore, to facilitate easy insertion and removal of the denture, the abutment devices were positioned in the same locations as in the previous denture, with careful attention paid to denture stability.

**Figure 5:**
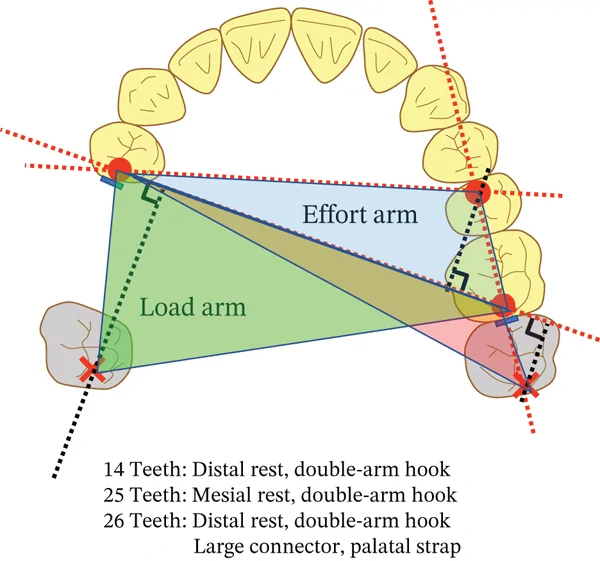
Design drawing for a maxillary denture. Set the position of the rest so that the forces are balanced, using the area of a triangle calculated with the abutment. Teeth 14: distal rest, double‐arm hook. Teeth 25: mesial rest, double‐arm hook. Teeth 26: distal rest, double‐arm hook. Large connector, palatal strap.

### 2.5. Neutral Zone and Flange Techniques

Given the significant resorption of the mandibular alveolar ridge, it was determined that measures were necessary to ensure stability by carefully positioning the artificial teeth. Therefore, the neutral zone technique was employed to determine the optimal arrangement of the artificial teeth [2, 3]. Using a working model of the mandible, a base plate was fabricated, and paper clips were placed at three locations. Then, using the Kerr compound (Envista Holdings Co., Tokyo, Japan), the area where the inward pressure from the cheeks and lips and the outward pressure from the tongue were balanced during oral function was determined. The neutral zone technique was performed on the mandible to record the equilibrium zone of the perioral muscles (Figure 6). Using the plaster core obtained from the neutral zone, GC Surpass (GC Corp., Tokyo, Japan) hard resin teeth were arranged. The occlusal pattern during lateral movement was designed to achieve bilateral balanced occlusion. Artificial teeth were arranged within the neutral zone to ensure denture stability (Figure 6). Furthermore, during the trial fitting, the flange technique [4] with a tissue conditioner was employed to obtain an impression of the base flange morphology reflecting the physiological movement of the surrounding muscles, thereby determining the shape of the denture base polished surface (Figure 7).

**Figure 6:**
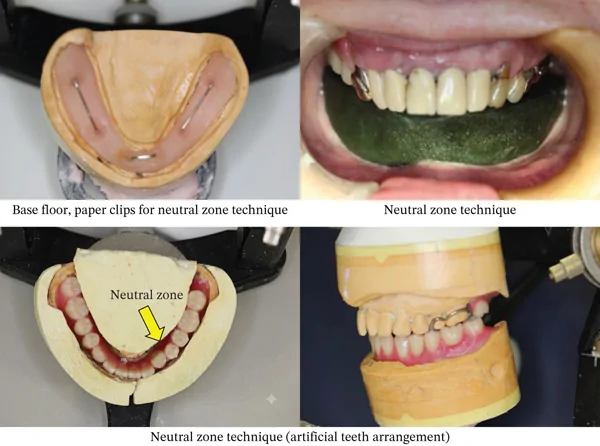
Neutral zone technique (artificial teeth arrangement).

**Figure 7:**
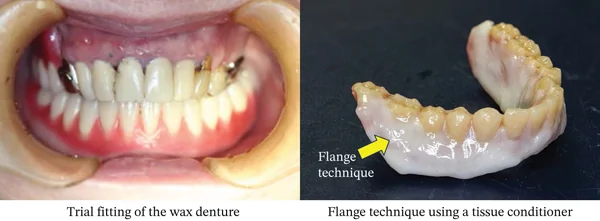
Flange technique.

## 3. Results

As a result of aligning the teeth within the neutral zone, it can be seen that the occlusion on the left side exhibits a crossbite. Furthermore, the stability of the lower denture is evident due to the shape of the denture base surface obtained using the flange technique. The patient′s chief complaints of chewing difficulties and food impaction were resolved, and satisfactory aesthetic and functional outcomes were achieved. Frontal, occlusal, and lateral views of the patient wearing the new dentures are shown (Figure 8).

**Figure 8:**
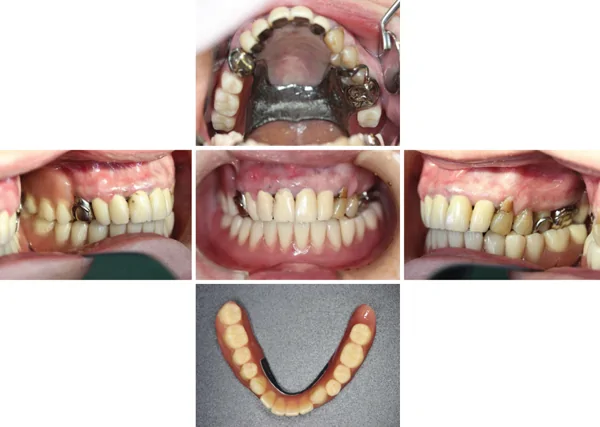
New dentures.

Masticatory function was evaluated following the fitting of new dentures using the Glucosensor GS‐II. The glucose concentration after the fitting of the new dentures was 206 mg/dL, indicating a favorable improvement in masticatory function.

### 3.1. Follow‐Up Assessment

The patient was regularly monitored at 1, 3, 6, and 12 months postinsertion. During the initial month, minor mucosal adjustments were performed twice on the lingual flange near the mylohyoid ridge due to slight localized pressure spots; however, no nerve compression symptoms or severe mucosal pain were reported. Beyond the 3‐month mark, no further structural adjustments were required. At the 12‐month recall, the denture remained highly stable without dislodgement during speech or mastication. Patient satisfaction was quantitatively and qualitatively confirmed to be maintained at a high level. Retesting masticatory performance at 12 months yielded a glucose concentration of 212 mg/dL, indicating sustained operational efficiency and full integration of the prosthesis within the patient’s neuromuscular dynamics.

## 4. Discussion

In prosthetic treatment for edentulous patients with severe alveolar ridge resorption, as seen in this case, the primary clinical challenge is establishing the three key elements of support, retention, and stability. Rehabilitation begins with a thorough assessment of the patient′s oral health status and remaining supporting tissues, where accurate impression techniques and jaw relation records are essential to ensure that the prosthesis can tolerate displacement forces [8]. Particularly in the mandible, where physical retention and the bony foundation′s area of support are extremely limited, it is essential to design a denture shape that harmonizes with the dynamics of the surrounding soft tissues. Furthermore, in patients with inadequate muscular coordination or those dissatisfied with conventional mandibular dentures, recording the neutral zone using functional impression materials helps effectively capture the musculature [8]. Although the application of the neutral zone technique is widely documented in the literature for edentulous rehabilitation, this specific case presented an unusually high clinical risk due to the confluence of severe Type V ridge atrophy and the proximity of the inferior alveolar nerve directly below the crestal mucosa. Placing teeth using standard anatomical landmarks in such cases routinely leads to vertical force concentration directly over the nerve path and dynamic dislodgement driven by hypermobile floor‐of‐mouth tissues. The primary innovation in this approach relies on the synchronized dual implementation of both the neutral zone and the flange technique to target two distinct physical planes simultaneously. Whereas the neutral zone technique establishes horizontal stability and proper coronal tooth arrangement within the equilibrium space, the flange technique customizes the polished surface in three dimensions to capture functional border sealing. Synergizing these two techniques ensured that lateral vector forces exerted by the buccinator and tongue muscles were converted into retentive forces rather than disruptive moments, preventing micromovements that would otherwise cause direct nerve trauma.

In edentulous prosthodontics, positioning artificial teeth within the neutral zone is a fundamental principle for achieving dynamic stability of the denture. In such cases, arranging artificial teeth on the conventional alveolar ridge crest tends to inhibit tongue movement or interfere with buccal muscle action, making the denture prone to elevation or overturning. In this case, by precisely recording the dynamics of the perioral muscles, by incorporating the patient′s individual functional space into the denture design, an environment was created in which the denture is “held in place” by muscular force rather than being “pushed back” by it during mastication or speech. This outcome is strongly supported by recent evidence demonstrating that neutral zone dentures offer significantly higher levels of patient satisfaction compared with conventional dentures across all functional aspects, including retention, stability, masticatory ability, speech, comfort, and esthetics [9, 10]. Furthermore, neutral zone approaches consistently improve oral health‐related QOL and posttreatment satisfaction in edentulous rehabilitations [11].

Cagna et al. [12] reported that while complete denture treatment for edentulous patients with severe alveolar ridge resorption is extremely challenging, achieving a physiologically optimal denture contour and proper denture positioning is necessary to maximize the stability, comfort, and function of the prosthesis; they further noted that the denture space recording method is highly effective as a complementary tool for this purpose. The flange technique is a representative method for recording denture space [4]. One of the patient′s chief complaints, “accumulation of food debris around the denture,” was caused by gaps forming between the denture margins and the mobile mucosa due to alveolar ridge resorption, resulting in inadequate marginal seal. By achieving optimal muscle harmony and border seal, the neutral zone approach effectively reduces food traps underneath the denture, directly addressing this common complaint. The flange technique, as applied here, uses a tissue conditioner during the artificial tooth arrangement trial‐fitting stage to modify the base margins and external shape through functional movements. This made it possible to impart a “dynamic thickness” to the denture base that cannot be fully captured by clinical impression‐taking alone. The thickened base margin makes close contact with the buccal mucosa, effectively preventing food from slipping under the denture and resolving the hygiene and discomfort issues associated with food debris accumulation. This demonstrates the advantage of imparting a functional form, which is difficult to achieve with conventional anatomical impression taking alone.

In this case, mechanical considerations regarding the maxillary partial denture cannot be overlooked, in addition to the design of the mandibular denture. In the maxilla, based on the theory of area balance connecting the abutment tooth lines, the rest positions were set over a wide area with the vertices of a triangle, thereby minimizing denture sinking and rotation. When a mandibular complete denture and a maxillary partial denture occlude, instability in one denture compromises the stability of the opposing denture as well. It can be said that the firm support provided to the maxilla resulted in a synergistic effect: Occlusal forces were transmitted to the mandible vertically and stably, thereby aiding in the retention of the mandibular denture.

Furthermore, long‐term follow‐up results demonstrate that combining these modalities promotes long‐term neuromuscular adaptation. The sustained high glucose concentration levels (206 mg/dL postfitting and 212 mg/dL at 12 months) confirm that functional recovery was not merely a transient postoperative improvement, but a stable long‐term outcome. The minimal need for postinsertion adjustments after 3 months underscores that functional modeling of the base effectively prevented chronic mucosal irritation and localized stress on the vulnerable inferior alveolar nerve region.

This case suggests that by achieving a trinity of occlusal surface morphology, support design, and harmony with soft tissues, it is possible to improve QOL even in difficult cases involving severe alveolar ridge resorption, without resorting to surgical intervention. As an assessment of masticatory function, the rise in glucose levels from 57 to 206 mg/dL also supports the conclusion of objective functional recovery. In particular, for elderly patients with osteoporosis or hypertension, maintaining good masticatory function is extremely important for nutritional intake. The technique used in this case does not require specialized equipment and can be performed using basic dental materials and a series of careful clinical steps. This approach is believed to have the potential to serve as a guideline for prosthetic treatment in a superaging society.

### 4.1. Limitations

Although the combined use of the neutral zone and flange techniques demonstrated significant clinical success in this case, several limitations should be noted. First, as a single case report, the findings cannot be generalized to all patients with severe mandibular ridge resorption. The degree of neuromuscular adaptability and anatomical variations, such as mucosal thickness and tongue activity, differ among individuals and may influence treatment outcomes. Second, although the 12‐month follow‐up provided valuable insight into medium‐term stability and patient satisfaction, longer‐term evaluation (e.g., 3–5 years) is necessary to assess progressive ridge resorption and long‐term wear of the artificial teeth. Finally, although masticatory function was quantitatively evaluated using the Glucosensor GS‐II, additional objective parameters—such as electromyographic analysis of the perioral muscles or maximum occlusal force measurements—could provide a more comprehensive understanding of the neuromuscular adaptation associated with these techniques. Further observational or prospective studies involving larger patient cohorts are warranted to validate these clinical findings.

## 5. Conclusion

In cases of severe resorption of the mandibular alveolar ridge, the combined use of the neutral zone technique and the flange technique proved highly effective in ensuring dynamic denture stability and improving marginal seal. This approach, which replaces functional pressure with form, has been shown to be a key factor in increasing the success rate of complete dentures in difficult cases.

## Author Contributions

H.A. contributed to the study as the first author and wrote the manuscript, and was responsible for conception, design, acquisition, analysis, and interpretation of data.

## Funding

No funding was received for this manuscript.

## Ethics Statement

This case report was conducted after the patient received detailed written and verbal explanations, written informed consent was obtained, and approval was obtained from the Ethics Committee of the School of Life Dentistry, Nippon Dental University. The ethical committee of Nippon Dental University approved the present study (NDU‐T2026‐7).

As this study involves humans and human data, I declare that it complies with the Declaration of Helsinki.

## Consent

Written informed consent was obtained from the patient for the publication of her personal and clinical information and images in this study.

## Conflicts of Interest

The author declares no conflicts of interest.

## Supporting information


**Supporting Information** Additional supporting information can be found online in the Supporting Information section. 
