# Supplementary material for: A Case Report of a Complete Removable Denture Designed to Restore Function in a Patient With Severe Resorption of the Mandibular Alveolar Ridge Using the Neutral Zone and Flange Techniques
Source: Case Rep Dent. 2026 Oct 13;2026:6174765. doi: 10.1155/crid/6174765 (PMC13659418; doi:10.1155/crid/6174765)
Supplement: Supplementary file 1 [file CRID-2026-6174765-s001.docx]

# CARE Checklist of information to include when writing a case report

**Topic	Item	Checklist item description	Reported on Line**

**Title	1**	The diagnosis or intervention of primary focus followed by the words “case report” . . . . . . . . . . . . . . . . . .	1page line 2-3

**Key Words	2**	2 to 5 key words that identify diagnoses or interventions in this case report, including "case report"	2page line 21-25

**Abstract**

**(no references)**

**3a**	Introduction: What is unique about this case and what does it add to the scientific literature?	2page line 2-7

**3b**	Main symptoms and/or important clinical findings . . . . . . . . . . . . . . . . . . . . . . . . . . . . . . . . . . . . . . . . . . . . . . . . . . .	2page line 8-10

**3c**	The main diagnoses, therapeutic interventions, and outcomes	3page line38- 4page line1

**3d**	Conclusion—What is the main “take-away” lesson(s) from this case?	7page line 27-30

**Introduction	4**	One or two paragraphs summarizing why this case is unique (**may include references**)	2page line 28-3page line5

**Patient Information	5a**	De-identified patient specific information	3page line 9-10

**5b**	Primary concerns and symptoms of the patient	3page line 10-15

**5c**	Medical, family, and psycho-social history including relevant genetic information	3page line 10-13

**5d**	Relevant past interventions with outcomes	3page line 9-10

**Clinical Findings**

**Timeline**

**Diagnostic Assessment**

**Therapeutic Intervention**

**Follow-up and Outcomes**

6 Describe significant physical examination (PE) and important clinical findings	3page line 13-25 Fig.1
7 Historical and current information from this episode of care organized as a timeline	3p line 9-15,4p line3-36

**8a**	Diagnostic testing (such as PE, laboratory testing, imaging, surveys).	3page line 16-35

**8b**	Diagnostic challenges (such as access to testing, financial, or cultural)	3page line 18-21

**8c**	Diagnosis (including other diagnoses considered)	3page line 38

**8d**	Prognosis (such as staging in oncology) where applicable	3page line 16-20

**9a**	Types of therapeutic intervention (such as pharmacologic, surgical, preventive, self-care) . . . . . . . . . . . . . . . . .	3page line 30-35

**9b**	Administration of therapeutic intervention (such as dosage, strength, duration)	4page line 3-4page 36

**9c**	Changes in therapeutic intervention (with rationale)	4page line 25-27

**10a**	Clinician and patient-assessed outcomes (if available)	5p line 1- line8

**10b**	Important follow-up diagnostic and other test results	5p line 11- 5p line 17

**10c**	Intervention adherence and tolerability (How was this assessed?)	5p line 6- 5p line 8

**10d**	Adverse and unanticipated events	5page line 1-3

**Discussion	11a**	A scientific discussion of the strengths AND limitations associated with this case report	7page line 8-18

**11b**	Discussion of the relevant medical literature **with references**	8page line11- line37

**11c**	The scientific rationale for any conclusions (including assessment of possible causes)	6page line37-7page line 6

**11d**	The primary “take-away” lessons of this case report (without references) in a one paragraph conclusion	7page line 21-24

**Patient Perspective	12**	The patient should share their perspective in one to two paragraphs on the treatment(s) they received . . . .	5page line 3-5

**Informed Consent	13**	Did the patient give informed consent? Please provide if requested . . . . . . . . . . . . . . . . . . . . . . . . . . . . . . . . . . . . . .	**Yes** **No**
